# Three-dimensional fiber orientation mapping of the human brain at micrometer resolution

**DOI:** 10.21203/rs.3.rs-4725871/v1

**Published:** 2024-08-07

**Authors:** Chao J. Liu, William Ammon, Robert J. Jones, Jackson C. Nolan, Dayang Gong, Chiara Maffei, Brian L. Edlow, Jean C. Augustinack, Caroline Magnain, Anastasia Yendiki, Martin Villiger, Bruce Fischl, Hui Wang

**Affiliations:** 1 Athinoula A. Martinos Center for Biomedical Imaging, Department of Radiology, Massachusetts General Hospital/Harvard Medical School, Charlestown, MA 02129, USA; 2 Center for Neurotechnology and Neurorecovery, Massachusetts General Hospital, Harvard Medical School, Boston, Massachusetts.; 3 Wellman Center for Photomedicine, Massachusetts General Hospital, Boston, MA, 02114, USA; 4 These authors contributed equally to this work

## Abstract

The accurate measurement of three-dimensional (3D) fiber orientation in the brain is crucial for reconstructing fiber pathways and studying their involvement in neurological diseases. Comprehensive reconstruction of axonal tracts and small fascicles requires high-resolution technology beyond the ability of current *in vivo* imaging (e.g. diffusion magnetic resonance imaging). Optical imaging methods such as polarization-sensitive optical coherence tomography (PS-OCT) and polarization microscopy can quantify fiber orientation at micrometer resolution but have been limited to two-dimensional in-plane orientation or thin slices, preventing the comprehensive study of connectivity in 3D. In this work we present a novel method to quantify volumetric 3D orientation in full angular space with PS-OCT. We measure the polarization contrasts of the brain sample from two illumination angles of 0 and 15 degrees and apply a computational method that yields the 3D optic axis orientation and true birefringence. We further present 3D fiber orientation maps of entire coronal cerebrum sections and brainstem with 10 μm in-plane resolution, revealing unprecedented details of fiber configurations. We envision that our method will open a promising avenue towards large-scale 3D fiber axis mapping in the human brain as well as other complex fibrous tissues at microscopic level.

## Introduction

The fiber tracts constituting the white matter in the human brain form an intricate network of connections that support neuronal communication and brain function. Deciphering the structural connectivity of the brain is crucial to understand normal behavior and what goes wrong in neurological disorders. Diffusion weighted magnetic resonance imaging (dMRI) detects the preferential direction of water diffusion, which is parallel to the fiber bundles; it allows fiber pathways to be imaged *in vivo* and has been widely used in clinical settings such as neurosurgical planning and targeting for deep brain stimulation ^1^. Despite tremendous advancements over the past decade ^2,3^, the mm-resolution of dMRI limits its ability to resolve complex fiber configurations, particularly in the regions where multiple fiber bundles from different directions intersect ^4^. Microscopic methods are necessary to unveil the ground truth fiber architectures and to guide the validation of new dMRI techniques ^4,5^. Optical methods have used various contrast mechanisms to image myelinated axons at micrometer resolution, such as fluorescent labeling ^6^, third harmonic generation ^7^ and anti-stokes Raman scattering ^8^. However, these techniques do not provide direct measures of fiber orientation that can be used to reconstruct white-matter tracts and study structural connectivity in the brain. Instead, fiber orientation is computationally derived from the acquired images, using techniques such as structure tensor analysis, which has been applied to histological staining ^9,10^, fluorescence microscopy ^11^, and label-free optical coherence tomography (OCT) images ^12^.

**Fig. 1:**
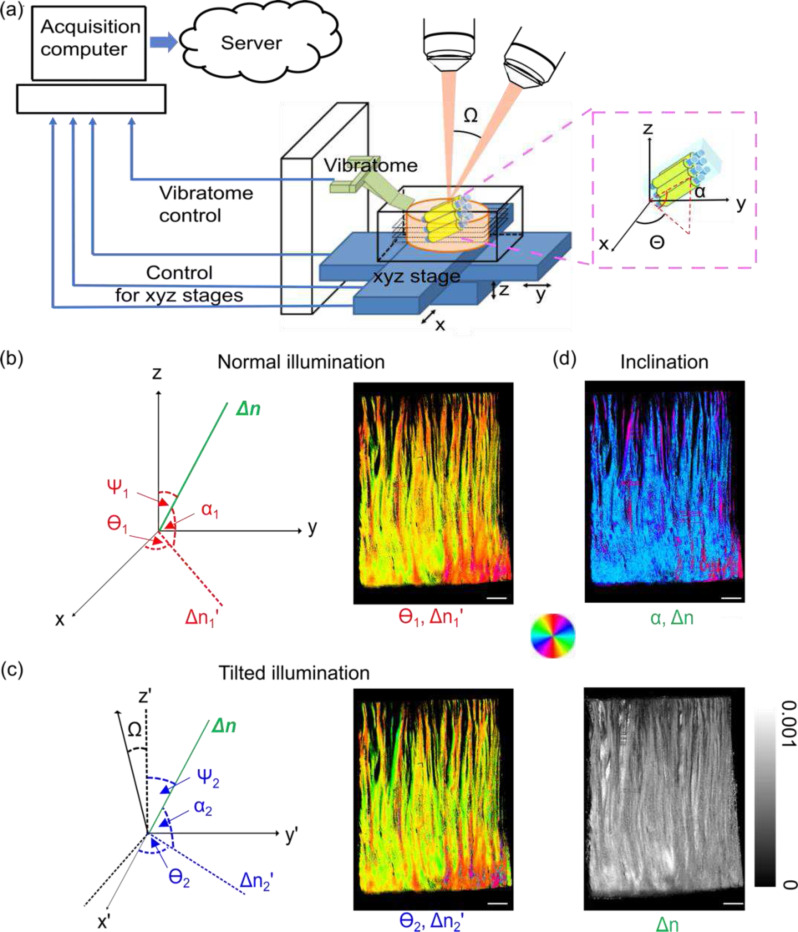
Computational optimization of true birefringence vector by normal and one tilted illumination. (a) System schematic. The insert shows the in-plane (Θ) and through-plane (α) orientations of fiber tracts. (b) The angle definitions in the normal illumination incidences (left) and the orientation images obtained from this incidence (${\text{θ}}_{1}$, right). (c) The angle definitions in the tilted illumination incidences (left) and the corresponding orientation images (${\text{θ}}_{2}$, right). (d) The computational retrieved inclination angle image (α, right). The angles of orientation in (b)-(d) are indicated by the color wheel. The brightness of the images is modulated by corresponding apparent birefringence $\Delta n_{1}^{\prime }$, $\Delta {n^{\prime }}_{2}$ and the true birefringence, Δn. The intensity of the true birefringence image is indicated by the scale bar in (d). Sclar bars: 1 mm.

Imaging techniques using light polarization take advantage of the birefringent nature of the myelin sheath surrounding axons to generate quantitative and label-free contrasts for myelinated fibers ^13^. Birefringence originates in the differential refraction between two orthogonal axes as light travels through the anisotropic materials. The fiber geometry causes optical anisotropy with negative birefringence and an optic axis orientation aligned with the physical fiber direction. In recent years, methods that rely on birefringence, such as polarized light imaging (PLI) ^14^ and automatic serial sectioning polarization-sensitive OCT (PS-OCT) ^15,16^, have been applied to map microscopic fiber orientation in large-scale brain samples. PLI uses multiple oblique views to derive the 3D fiber orientations in human brain sections, after cutting of the sample into 50–100 μm thick slices ^17^. Following fiber tracts across sections requires inter-slice registration with microscopic accuracy, which is an extremely challenging task ^17,18^. In serial sectioning PS-OCT, the blockface of the sample is imaged prior to sectioning, thus avoiding section-to-section distortions. The conventional optic axis orientation measurement of PS-OCT from one illumination angle quantifies an in-plane fiber orientation (Θ, Fig 1a), which is a projection of the 3D fiber axis onto a plane orthogonal to the beam. Information on the through-plane fiber orientation (α, Fig 1a) is not captured. However, fiber tracts in the brain run in all different directions in 3D; therefore, it is impossible to track them without the full 3D axis orientation. Another drawback of traditional PS-OCT is that it measures an apparent birefringence that has a dependence on the through-plane axis. Without removing the dependency, the measured birefringence does not truly reflect the structural anisotropy, which is an important indicator of myelin integrity in neurological disorders ^19^. One approach to obtain the through-plane orientation is to use multiple illumination angles. The in-plane optic axis orientations obtained by different illumination angles allow the calculation of the through-plane orientation via a geometric derivation. Our previous study suggested a minimum of three beams were required to accurately recover the 3D orientation of muscle fibers ^20^. Similarly, various illumination configurations have been proposed in PS-OCT and validated in simple birefringent samples of tendons and muscles ^20–23^. Those configurations have been using widely spaced imaging directions, and either excluding or assuming optic axes co-planar to the illumination beam directions, therefore limiting the range of the measurable through-plane axis. Furthermore, 3D fiber orientation measurements using PS-OCT in the human brain have not been reported.

In this work, we present the estimation of 3D axis orientation in human brain tissue that allows a complete mapping of the fiber axis within the 360-degree spherical space with a novel procedure and a computational framework. The estimation is enabled by only two illumination beams separated by a small angle of 15 deg. This approach formulates a birefringence vector composed of both optic axis orientation and true birefringence, which is estimated by minimizing the error from the two measured birefringence values and in-plane optic axis orientations. We conduct a systematic validation on the performance of our approach and show the 3D fiber orientation maps in various large human brain samples, including coronal sections of a whole hemisphere and a medulla sample.

## Results

### Reconstruction of 3D axis orientation via two illumination angles

Traditional PS-OCT only measures the apparent birefringence and in-plane optic axis orientation, whereas the through-plane component of the 3D axis is lost. Recent PS-OCT work has reported that the true 3D optic axis can be recovered by the measurements of apparent birefringence ^21^ or optic axis orientations ^20^ at variable illumination angles. Both the measured apparent birefringence and the optic axis have a dependence on the illumination angle and the measurements from different incident angles are linked by the through-plane orientation of the fibers. Geometrically, the 3D optic axis lies along the intersection of multiple planes, each of which is formed by an incident beam and its corresponding optic axis orientation. However, it is necessary to also account for the effective birefringence values, in order to manage the case when the optic axis is co-planar to the illumination beams, and hence the two planes coincide. Our forward model uses both optic axis and birefringence to capture these physical relationships.

To estimate the 3D optic axis via two illuminations, we first define the true birefringence vector $\text{Δ}n=\text{Δ}n[cos\text{Θ}cos\alpha \;\sin\text{Θ}cos\alpha \;sin\alpha {]}^{T}$, where Δn is the true birefringence, Θ is the in-plane orientation, and α is the through-plane orientation (see the insert in Fig. 1a). We imaged the sample with normal illumination and illumination tilted by Ωdeg about the y-axis with our automatic serial sectional PS-OCT system (Fig. 1a and see Methods for details). Under the normal illumination, we obtained the apparent birefringence $\Delta n_{1}^{\prime }$ and orientation measurement ${\text{Θ}}_{1}$ (Fig. 1b); whereas under the tilted illumination, we measured the apparent birefringence $\Delta n_{2}^{\prime }$ and orientation measurement ${\text{Θ}}_{2}$ (Fig. 1c). By defining estimated birefringence vectors $\left(\text{Δ}n_{1,2}^{\prime }\right)$ the two illuminations, we formulated the optimization problem in Eq. 1 as finding the birefringence vector Δn that minimizes the sum of the mean square errors between the estimated and the measured apparent birefringence vectors under the two illuminations (see Methods for details):

(1) $$\underset{\tilde{\Delta n}}{\min}\left({\left\|\tilde{\text{Δ}n_{1}^{\prime }}-\text{Δ}n_{1}^{\prime }\right\|}_{2}^{2}+{\left\|\tilde{\text{Δ}n_{2}^{\prime }}-\text{Δ}n_{2}^{\prime }\right\|}_{2}^{2}\right).$$

After estimating the true birefringence vector Δn, we then obtained the true birefringence Δn and through plane orientation α (Fig. 1d). The data acquisition and image reconstruction procedures are demonstrated in Fig. 1, using a homogeneous fibrous sample in the human brain.

### Recovering full-range through-plane axis orientation with high accuracy

**Fig. 2:**
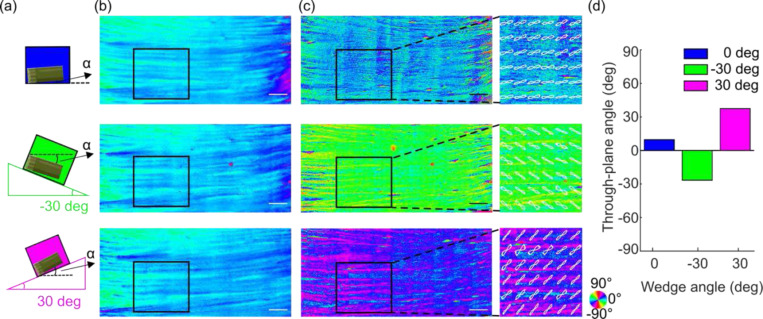
Characterization of through-plane orientation measurement for different inclination angles. (a) A cartoon illustrating the corpus callosum sample positioned on 0 deg (no wedge), − 30 deg wedge, and 30 deg wedge to create different inclination angles. α: inclination angle. (b) The in-plane orientation images of the sample on three wedge scenarios. (c) The through-plane orientation images of the sample on three wedge scenarios. The enlarged boxes of the ROI show the FODs overlaid on the orientation image. The angles of orientation in (b) and (c) are indicated by the color wheel. (d) The average through-plane orientation of the FODs within the ROIs in (c). Scale bars: 1 mm.

We used a section of a human corpus callosum in which the fibers run in a mostly uniform direction to validate the accuracy of the 3D fiber orientation estimation. To create different through-plane angles, we positioned the sample on a horizontal surface (0 deg) and two triangular wedges made of agarose gel (−30 and 30 deg, Fig. 2a), respectively. The long axis of the fibers was positioned along the x-axis of the laboratory frame, creating a 0 deg in-plane orientation. The obtained PS-OCT measurements were obtained from the normal and a 15 deg tilted illuminations in each experimental condition, and 3D axis orientation was estimated using the proposed method above. As expected, our optimization yielded the same estimated in-plane orientations of 0 deg (Fig. 2b), whereas distinct through-plane orientations with a difference of approximately 30 deg between conditions 1 and 2, and 1 and 3 (Fig. 2c). We further examined the through-plane orientation in a smaller region of interest (ROI) and plotted the fiber orientation distributions (FOD). We found the mean of the through-plane FODs to be 9.2 deg on 0 deg surface (no wedge), −26.9 deg on −30 deg wedge and 37.7 deg on 30 deg wedge (Fig. 2d). The difference between the three estimates agreed with the known difference between the inclination angles of the wedges. The estimates showed a positive offset consistently with respect to the preset wedge angles, indicating the presence of the fibers oriented at a small through-plane angle at the 0 deg wedge setting (about 6.7 deg on average). It is important to underline that our method was able to distinguish the positive and negative sign of the through-plane orientations (green vs. magenta wedges in Fig. 2), which is critical in connectivity analysis as 30 deg and −30 deg fibers run into completely different directions.

**Fig. 3:**
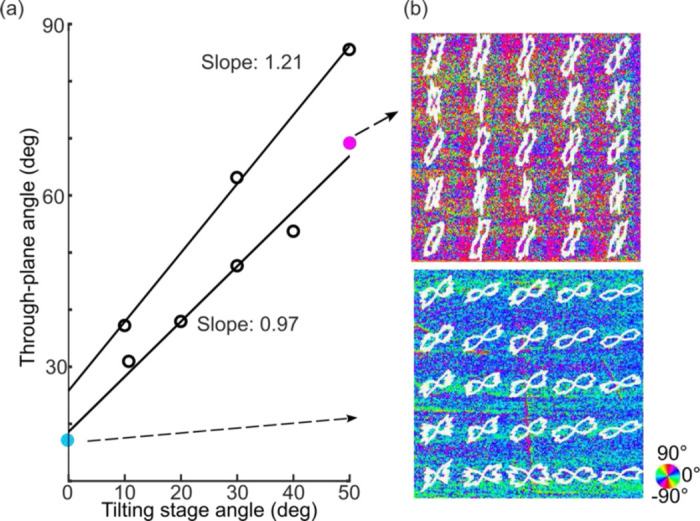
Characterization of the range of through-plane orientation estimation. (a) Two independent through-plane orientation measurements at different tilting stage angles. (b) Representative images of through-plane orientation when the tilting stage was set at 0 deg and 50 deg, with FODs overlaid. The angles of orientation are indicated by the color wheel. The sample size in (b) is 2.5mm × 2.5mm.

We then used another block of the corpus callosum sample to characterize the range of our through-plane orientation measurement. As the white matter fiber tracts in the brain could run in all directions in spherical space, it is important for our method to capture the full +/−90 deg range of through-plane angles. We performed two experiments on different regions of the sample. The sample was positioned on a tilting stage to artificially vary the through-plane angle. In the first experiment, the stage was tilted counterclockwise from 0 deg to 50 deg in 10 deg steps. Our estimated through-plane orientation successfully followed the preset tilting angles (Fig. 3a). A linear fitting of the estimated through-plane orientation with respect to the preset tilting angle revealed a slope of 0.97, very close to the theoretical value of 1. As an example, we showed two through-plane orientation maps reconstructed as the stage was positioned at 0 deg and 50 deg, in Fig. 3b. The mean through-plane orientation of the fibers was measured to be 18.6 deg and 73.6 deg, respectively. In the second experiment, we imaged another region of the sample by tilting the stage at 10 deg, 30 deg and 50 deg angles and estimated the corresponding through-plane orientation. The slope of a linear fitting between the estimation and the pre-set tilting angle was 1.21, and the estimated through-plane orientation at 0 deg tilting was 26.8 deg. The slight deviation of the fitted slope from 1 was possibly attributed to the increased noise at the large tilting angle, as the estimated through-plane orientation was close to 90 deg. Nevertheless, we demonstrated that our method can measure a full range of through-plane orientation angles. Taken together with Fig. 2, which shows a successful estimation of the sign of the through-plane orientation, these results suggest that our method is capable of recovering the full spherical range of the 3D fiber orientation in the human brain.

### Robust estimation under variety of in-plane orientations

**Fig. 4:**
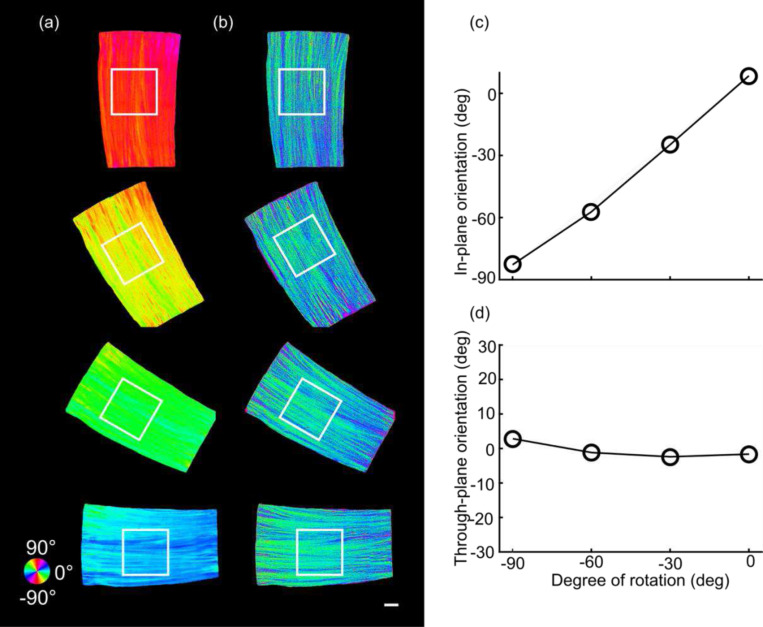
Characterization of through-plane orientation measurement at four different in-plane orientation angles on a corpus callosum sample. (a) The estimated in-plane orientation images at in-plane rotations of −90 deg, −60 deg, −30 deg, and 0 deg. (b) The corresponding through-plane orientation images. The black boxes indicate the ROIs to obtain the FODs. The angles of orientation in (a) and (b) are indicated by the color wheel. The average in-plane (c) and through-plane (d) orientation of the FODs within the ROIs shown in (a) and (b) with respect to the degree of rotation, separately. Scale bar: 1mm.

A previous study showed that the accuracy of the through-plane axis was highly dependent on the in-plane orientation; as a result, a minimum of three illumination angles was required to recover the through-plane axis reliably ^20^. The method that we propose here uses an optimization algorithm that eliminates the need for the third illumination angle, while retaining a viable through-plane orientation estimation. To validate the robustness of the estimation under a variety of in-plane orientation scenarios, we mounted a corpus callosum sample on a horizontal surface while rotating it counterclockwise from 0 to 90 deg in the XY plane by 30 deg steps. We expected that through-plane orientation should remain constant around 0 deg while the in-plane orientation should increase by 90 deg through rotation. We obtained the measurements via the two illumination angles, normal and tilted by 15 deg (Fig. 4). Our method estimated the in-plane orientations accurately (Fig. 4a) when the sample was oriented at −90, −60, −30 and 0 deg in the XY plane. Meanwhile, the through-plane orientation estimate was consistent among the different in-plane orientation setups (Fig. 4b). To evaluate our method quantitatively, we analyzed the fiber orientation distributions (FODs) of a 3 mm × 3 mm ROI as shown in Fig. 4a and Fig. 4b. The mean estimated in-plane orientation was −82.8, −57.2, −24.8 and 8.5 deg (Fig. 4c), accurately following the in-plane rotation. Note that there was a small angular offset (average of 5.9 deg) between the long axis of the fibers and the x-axis of the laboratory frame. The through-plane orientation estimation remained consistently close to 0 deg (2.8, −1.2, −2.4 and −1.6 deg) across the different in-plane orientation settings (Fig. 4d). This suggests that our method with only two illumination incidences can measure the 3D axis of all fibers robustly regardless of their orientation in the XY plane.

We demonstrated the advantage of a strategy using an xy-axis swap at 45 deg for through-plane axis estimation (Fig. 4, see details in Methods), by comparing to the results without xy-swap (using Eq. 1, 2, 3 for all in-plane orientations). In the latter case, both the in-plane (Fig. S2a) and through-plane (Fig. S2b) orientation showed high errors at −90 deg rotation (see Fig. S1 for details). We also compared our optimization result against an earlier method using a geometric derivation (Fig. S2c, see details in reference ^16^). Using only one tilted beam about the y-axis, the geometric derivation method resulted in errors in through-plane estimation as the in-plane angle reached −60 deg. The error continued to grow as the in-plane angle increased to −90 deg.

### Estimating true birefringence of myelin content

**Fig.5:**
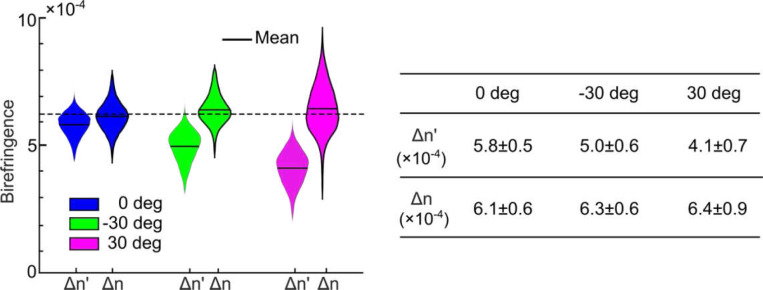
Estimation of birefringence of a corpus callosum sample with different inclination angles. The violin plots of average apparent $\left(\Delta n^{\prime }\right)$ and true birefringence Δn within the ROIs in Fig. 2(c). The mean value of the true birefringence (expected value) is indicated by the dashed black line. The $\Delta n^{\prime }$ and Δn values are indicated in the table as mean ± standard deviation within the ROIs.

Birefringence is a measure of myelin concentration in the brain and has important indications in neurodegenerative disorders involving myelin degradation. However, it is known that traditional PS-OCT measures an apparent birefringence that depends on the optic axis orientation of the tissue: the larger the through-plane orientation (α), the smaller the apparent birefringence $\left(\text{Δ}n^{\prime }\right)$^14^. This is in contrast to true birefringence (Δn), which depends only on structural anisotropy and not axis orientation. Estimation of true birefringence requires removing the effect of the through-plane angle. Using our computational framework, we also investigated the estimation of true birefringence in the corpus callosum sample. We computed the mean of the apparent birefringence and true birefringence within the ROIs from the three inclination setups as shown in Fig. 2a and 2b. As expected, the apparent birefringence was reduced in the ±30 deg inclination setups (4.9 × 10^−4^ and 4.1 × 10^−4^) compared to the 0 deg setup (5.8 × 10^−4^), showing the dependency on the through-plane angle (Fig. 5). By jointly estimating the through-plane angle and true birefringence, we found that the latter remained consistent across the three setups, regardless of the through-plane angle (mean value: 6.2 × 10^−4^), thus providing an unbiased measure of the underlying myelin content in this corpus callosum sample.

### Mapping 3D fiber orientation of a full coronal section in the human brain

**Fig. 6:**
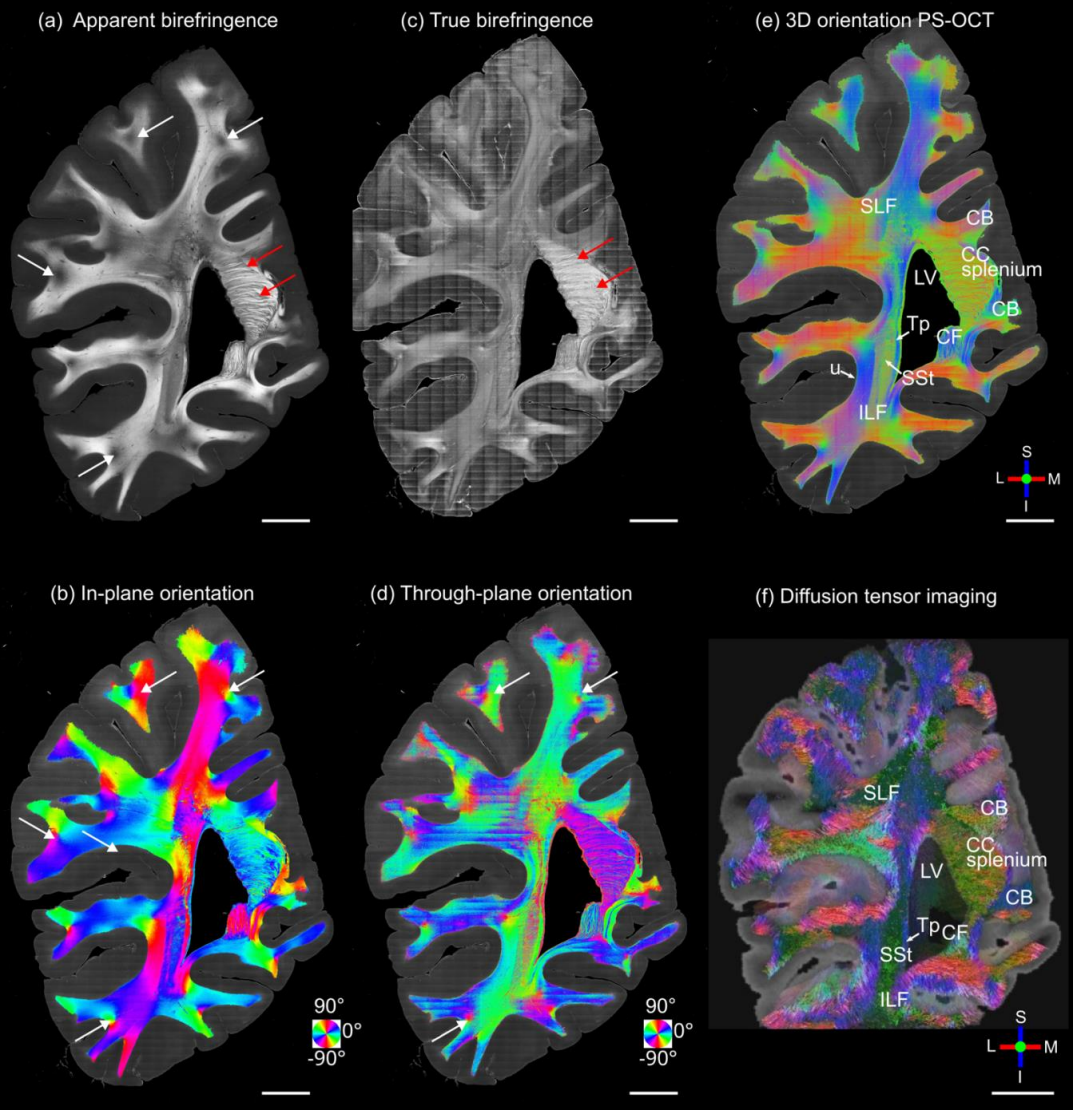
3D fiber orientation maps of the coronal slice sample: (a) apparent birefringence, (b) in-plane orientation, (c) true birefringence, (d) through-plane orientation, (e) 3D orientation from PS-OCT, and (f) 3D orientation from diffusion MRI. Red arrows in (a) and (c) indicate some fibers bundles in the splenium of the corpus callosum. White arrows in (a), (b), and (d) indicated some fibers bundles beneath the sulcus region. The in-plane and through-plane orientations are color-coded as indicated by the color wheels in (b) and (d). 3D orientations are color-coded as indicated by the red (left/right), green (anterior/posterior) and blue (inferior/superior) orthogonal axes in (e) and (f). Scale bars: 10 mm. Several anatomical structures are labeled in (d) and (e): LV, lateral ventricle; CC splenium, splenium of the corpus callosum; Tp, tapetum; SSt, sagittal stratum; ILF, inferior longitudinal fasciculus; u, u-fibers. L: lateral; M: medial; S: superior; I: inferior.

To investigate the feasibility of 3D orientation measurements in a large-scale sample, we imaged a coronal section of a human brain hemisphere, with a surface area of ~6 cm × 9 cm. We first acquired dMRI data of the sample followed by PS-OCT. For PS-OCT imaging, the tissue surface was flat-faced, and imaged with both normal and 15 deg tilted illuminations to recover the 3D orientations. We generated two maps of apparent birefringence and in-plane orientation obtained with traditional single-illumination PS-OCT (Fig. 6a-6b), and three additional maps acquired with our method, including true birefringence, through-plane orientation and 3D axis orientation (Fig. 6c-6e). As a comparison, we also show dMRI tractography in the same plane as the PS-OCT images (Fig. 6f).

Overall, the 3D orientation map of PS-OCT (Fig. 6e) is consistent with dMRI (Fig. 6f) and the known neuroanatomy of white matter architecture in this region (Fig. 6) ^24^. However, the much higher resolution of PS-OCT can be easily appreciated in the images. In the 3D orientation map, medial-lateral/right-left (red) and inferior-superior (blue) directions are in-plane and anterior-posterior (green) is through-plane. The splenium of the corpus callosum is situated on the superiomedial border of the lateral ventricle and contains highly birefringent fibers oriented in the anterior-posterior (green) and medial-lateral (red) directions. Lateral to the lateral ventricle, three white matter tracts could be clearly delineated. The tapetum (Tp) borders the lateral edge of the lateral ventricle and contains fibers coursing inferior-superior (blue). The sagittal stratum (SSt) sits lateral to the Tp and contained fibers running primarily anterior-posterior (green). Lateral to the SSt, the inferior longitudinal fasciculus (ILF) consists of mostly inferior-superior oriented fibers (blue). Throughout the superficial white matter, short association fibers (e.g., U-fibers), are seen wrapping around the sulci and extending down the gyri into gray matter. Gyral fibers show predominantly in-plane orientations (red, blue), while small bundles of through plane fibers (green) are consistently observed at the base of sulci. The high-resolution 3D fiber orientation map of PS-OCT depicts a diverse, heterogeneous organization of fiber bundles in deeper white matter. The smooth transition of the colored fiber orientation along tracts indicates realistic continuity that one might expect in the densely packed fascicular highways of the deep white matter.

Compared to traditional, apparent birefringence, true birefringence revealed the unbiased myelin distribution in the brain by removing the effect of the through-plane angle. In the apparent birefringence maps, dark spots were consistently present in fibers right beneath sulci (white arrows in Fig. 6a), where multiple colors in the in-plane orientation map (Fig. 6b) indicated the confluence of fibers bundles from different cortical regions into the superficial white matter. This low apparent birefringence is not due to low myelin content. Instead, it is caused by a group of fibers going through plane, proved by the high through-plane angle at the same locations (Fig. 6d). These dark spots are diminished in the true birefringence map (Fig. 6c). It is also intriguing to find that the splenium of the corpus callosum exhibits much higher true birefringence compared to the rest of the white matter in this section (red arrows in Fig. 6c), as effect that is confounded in the apparent birefringence map by the high through-plane angles.

### Volumetric 3D orientation and tractography of a human brainstem

**Fig. 7:**
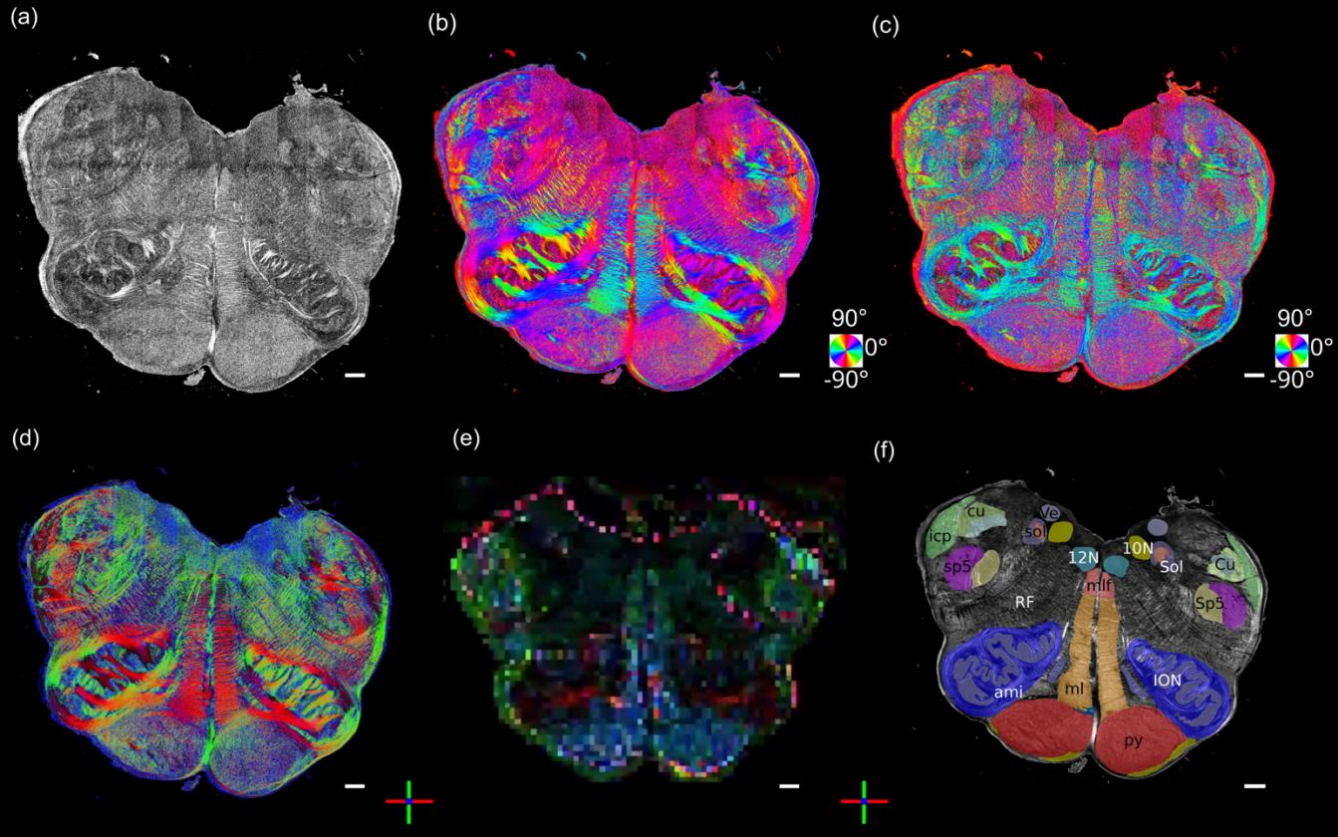
3D fiber orientation maps of a brainstem section. (a) True birefringence, (b) in-plane orientation, (c) through-plane orientation, (d) color rendering of the 3D orientation, (e) 3D orientation of co-aligned dMRI image, and (f) segmentation based on the Paxinos atlas. The in-plane and through-plane orientations are color-coded as indicated by the color wheels in (b) and (c). 3D orientations are color-coded as indicated by the red (left/right), green (anterior/posterior) and blue (inferior/superior) orthogonal axes in (d) and (e). Scale bars: 1mm. Abbreviations of anatomical structures: 10N, vagus nerve nucleus;12N, hypoglossal nucleus; ami,amiculum of the olive; Cu, cuneate nucleus; cu, cuneate fasciculus; icp, inferior cerebellar peduncle; ION, inferior olivary nucleus; ml, medial lemniscus; mlf, medial longitudinal fasciculus; py, pyramidal tract; RF, reticular formation; Sol, solitary nucleus; sol, solitary tract; Sp5: spinal trigeminal nucleus; sp5, spinal trigeminal tract; Ve, vestibular nucleus.

The micrometer resolution PS-OCT reveals complex configurations of small fiber tracts at a voxel size 5,000–1,000,000 times smaller than the dMRI. Particularly in the brainstem, compact fiber tracts that connect small nuclei run in all directions, making it extraordinarily challenging to resolve small fibers (Fig. 7f). To demonstrate the advantages of measuring 3D axis orientation by PS-OCT, we performed volumetric imaging of a slab of a human brainstem at the level of the medulla oblongata (Fig. 7a-7d), following dMRI scanning of the whole brainstem and cerebellum (Fig. 7e). The tissue went through PS-OCT imaging with both normal and 15 deg tilted illumination and sectioning as previously reported for large volumetric reconstruction ^17^. The total reconstructed volume was approximately 30×20×3 mm^3^. The brainstem was positioned in an axial plane, i.e., left-right (red), and anterior-posterior (green) being the x and y axis of our laboratory frame, respectively. It is well known that there are large numbers of fibers running through the axial plane in the brainstem, as well as substantial in-plane fibers connecting the small nuclei. The true birefringence map indicated a high myelin content in the human medulla with the presence of various nuclei. Using the maps generated by PS-OCT, we annotated some of the major structures on the high-resolution images based on the Paxinos Atlas (Fig. 7f) ^25^ and inspected the orientation of the fiber tracts (Fig. 7b-7d). For example, the direction of internal arcuate fibers seen in the reticular formation (RF) running from the cuneate (Cu) and gracile nuclei (not shown in Fig. 7) to the contralateral medial lemniscus (ml), is consistent with the mostly in plane fibers in the RF and ml. The amiculum of the olive (ami) is a capsule of myelinated fibers surrounding the inferior olivary (IO) running both in-plane and through-plane. The spinal trigeminal tract (sp5), as well as the inferior cerebellar peduncle (icp), run inferior to superior, connected with other structures via numerous input and output fibers throughout their length in the axial plane. Finally, two other main fiber tracts, the pyramidal (pyr) and solitary tract (sol) run inferior to superior and thus appear mostly blue in the color rendering of the 3D fiber orientation.

**Fig. 8:**
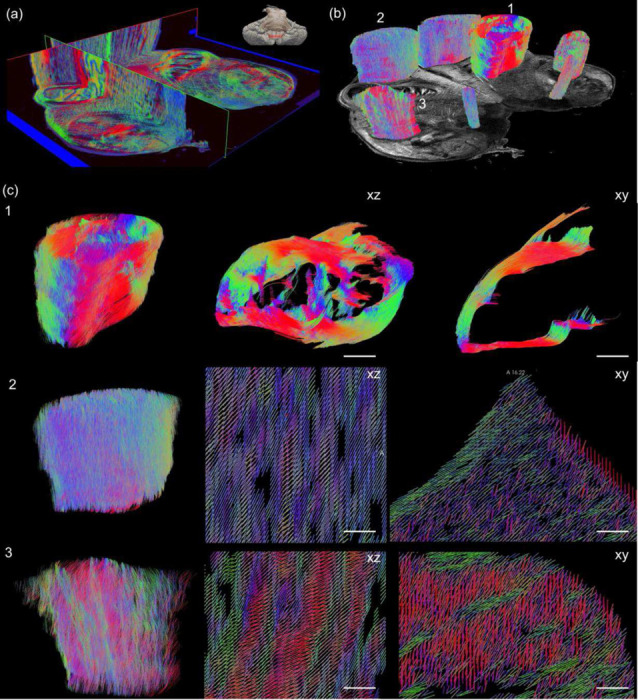
Volumetric rendering of 3D axis orientation and tractography of human medulla by PS-OCT. (a) The 3D axis orientation maps of fiber tracts are presented in 3D orthogonal views. The inset indicates the anatomical location (red band) of the medullar slab in the brainstem. (b) Tractography obtained on selective ROIs based on 3D axis orientation. ROI 1: amiculum of the olive (ami); ROI 2: pyramidal tract (py); ROI 3: spinal trigeminal tract (sp5). (c) Zoom in tractography (ROI 1, and left panel of ROI 2 and 3) and orientation vectors (middle and right panels of ROI 2 and 3) showing detailed fiber trajectory in three ROIs as indicated in (b). 1: side (left) and top (middle) view of the ami and branching of fiber bundles (right) through a small ROI (sphere in the middle). 2 and 3: volumetric rendering (left), side view (middle) and top view (right) of py and sp5. Scale bars: 250 μm.

We demonstrated the advantage of volumetric imaging with our method by reconstructing 19 consecutive block-face sections with a 10 μm in-plane resolution and 150 μm slice thickness. The 2.85 mm volumetric rendering of true birefringence (Supplementary Video 1), in-plane orientation (Supplementary Video 2), through-plane orientation Supplementary Video 3), and 3D axis orientation (Fig. 8a) present the sophisticated fiber network in the human brainstem. The geometry of the fiber tracts was very well preserved without the errors caused by inter-slice alignment and the slice damage typically seen in traditional histological images including PLI. To leverage the volumetric 3D axis orientation for connectivity studies, we tracked the trajectory of the fibers using off-the-shelf dMRI tractography toolkit in selective fiber bundles (Fig. 8b), including the solitary tract (sol), the amiculum of the olive (ami, ROI 1), the pyramidal tract (py, ROI 2), and the spinal trigeminal tract (sp5, ROI 3). We then visualized the zoomed tracts in the latter three ROIs. The ami forms a circumferential layer surrounding the inferior olivary nucleus (ION) while intervening with the ION from different directions. It is interesting to see that complex fiber configurations occur throughout white matter regions. For example, the fibers in py and sp5 form a twisting and inter-weaving pattern in small bundles while they travel through the superior-inferior axis. Moreover, the bundles in py and sp5 reveal differed inter-weaving angles that are smaller in py than sp5. Those twisting patterns might facilitate a large number of axonal groups reaching their widely spread terminals through these compact white matter pathways and forming a certain topology in their trajectories. The microscopic details revealed by our technique can provide unprecedented information to understand the complicated fiber connections in the brainstem that play a central role in functions such as modulating wakefulness in human consciousness ^26^.

## Discussion

Directly imaging connective pathways in the human brain is crucial for neurosurgical planning and intraoperative guidance. In this work, we presented a new approach for estimating 3D fiber orientation and true birefringence of human brain samples using PS-OCT, enabling investigation of connectivity and myelin content at the microscale. Traditional PS-OCT only measures a projection of 3D fiber orientation and an apparent birefringence that depends on through-plane angle of fiber tracts. Our method measures fiber orientations in full spherical orientation space and birefringence free of the orientation bias to reveal the true structural anisotropy of myelinated fibers. By formulating a birefringence vector composed of true birefringence value and 3D optic axis orientation vector, we verified that our method could recover accurate estimates of a large range of through-plane angles at different in-plane orientations. We showed 3D fiber orientation maps in full sections of a human cerebral hemisphere and a brainstem. The 10 μm resolution maps revealed intricate fiber organization far beyond the capabilities of non-invasive imaging techniques such as dMRI. To our knowledge, this is the first thorough characterization of 3D fiber axis measurement in the human brain with microscopic spatial resolution, using PS-OCT based methods.

Our major contribution is the accurate and robust reconstruction of 3D axis orientation in full spherical space, using only two illumination beams. Previously, quantifying 3D orientation by PS-OCT via multiple beams has been proposed based on either apparent birefringence deviation or geometrical transformation of in-plane orientations ^20,21^. A common caveat of those methods is that they did not show the estimation for the full angular space, a critical factor for studying structural connectivity in the human brain as the fiber tracts run in all different directions. Early studies suffered from the limitation that only the axis lying within the 2D plane spanned by the illumination beams could be recovered ^21^. Later methods removed this limitation, but required a third illumination to complete the estimation in the 3D space, substantially increasing the acquisition time and hardware complexity ^20,22^. The birefringence vector defined in this work makes use of both apparent birefringence and in-plane orientation metrics in PS-OCT. This formulation obviates the requirement for the third illumination angle and allows the retrieval of the vector from only two sets of measurements. This implementation significantly reduces the engineering complexity and the acquisition time for large-scale samples. Another work utilized a dual-angle PS-OCT with a large tilt angle, based on geometric reasoning but failed in our hands for axes co-planar to the two illumination beams ^23^. Here we significantly advanced the 3D axis orientation reconstruction with a small tilt angle of 15 deg as the second illumination, which reduces the intensity loss of light caused by refraction and yields a high signal-to-noise-ratio (SNR). The small tilting angle also simplifies image registration between the illuminations. Importantly, we showed that our method was able to recover the axis orientation in full spherical space (Fig. 2–4) with high angular precision (Fig. 3). Therefore, the proposed PS-OCT method enables quantitative characterization of comprehensive fiber configurations in the human brain.

Signal-to-noise-ratio (SNR) plays an important role in 3D orientation estimation.Especially in *ex vivo* human tissue imaging, the long fixation time significantly increases scattering, and therefore reduces the SNR and the degree of polarization over depth. In our procedure, we applied refractive index matching to our samples to increase the SNR in the PS-OCT measurements. Previously, we showed that index matching significantly improved the SNR and thus enhanced the accuracy of in-plane orientation and apparent birefringence measurements ^27^. It is noted that the index matching procedure may not be necessary in *in vivo* tissue imaging, where scattering is much smaller. Previous studies using fresh or lightly fixed birefringent tissue have demonstrated success of 3D axis measurement without refractive index matching.

Imaging connectional anatomy at the microscopic level is imperative for understanding the human brain. Although 3D fiber orientation has been previously computed from images of histological stains or measured with polarized light imaging, these approaches involve registration of a stack of 2D brain sections to recover volumetric structure, a challenging task due to the nonlinear distortions induced by sectioning and tissue mounting ^28^. This renders continuous tracing of 3D fibers across sections at microscopic scale impractical ^4^. In this work, we can achieve this by incorporating a serial sectioning PS-OCT technique ^15^ into 3D axis imaging, whose unique advantage is that the blockface of the tissue is imaged before it is cut, therefore eliminating tissue distortion and preserving volumetric inter-slice alignment with microscopic accuracy ^28^. We showed examples of 3D axis orientation maps in volumetric reconstructions of brainstem (Fig. 6–8), revealing the extraordinarily complex organization of fiber bundles at microscopic scale, including twisting and inter-weaving with different angles. Importantly, the reconstructed volume preserves the integrity of fiber pathways in 3D without aforementioned distortions. It is noted that, in the current study, we did not perform tractography for the entire brainstem sample, as standard dMRI tractography tools are not suitable for the full complexity of fiber architectures at the microscale. Future development involves building dedicated tools for microscopic tractography. Further development of serial PS-OCT and computational tools opens the potential of large-scale neuroanatomical connectivity studies at unprecedented details.

Diffusion MRI has been widely used in clinical and preclinical studies. However, the millimeter resolution of dMRI is insufficient in regions where multiple microscopic fascicles converge, forming complicated structures like crossing, branching, or fanning ^29^; microscopic imaging needed to validate and to guide dMRI technologies in those challenging regions ^4,5^. PS-OCT has been used to evaluate the accuracy of dMRI orientation obtained with different q-space sampling schemes, spatial resolutions, and orientation reconstruction methods in human white matter samples ^30,31^. The ability to measure 3D axis orientation in large-scale samples will enable full validation of dMRI.

Birefringence has been used as a biomarker in myelin involved neurological disorders, such as multiple sclerosis ^32^, stroke ^33^, cerebral amyloid angiopathy ^34^, and Alzheimer’s disease ^35^. Previous studies have found myelin defects in those diseases and an associated birefringence reduction. The alteration of birefringence is an indication of changes in the integrity and alignment of myelin sheaths. One drawback of traditional PS-OCT is that it measures an apparent birefringence that depends on the fiber orientation and hence may yield biased estimates of myelin content (Fig. 5). In this work, we introduced true birefringence, which is free of the influence of through-plane fiber orientation and hence yields an unbiased measure of myelin content (Fig. 5). The results from the whole coronal section showed a significantly higher true birefringence in corpus callosum, indicating a high myelin content, which is supported in previous studies using different imaging modalities (e.g., see red arrows in Fig. 6). Our method to estimate the birefringence potentially advances our understanding of myelin distribution in the brain, and hence merits further investigation and validation. It is worthwhile mentioning that the birefringence estimation is also dependent on the objective used in the microscope that depicts different scales of myelin arrangement ^36^. With similar resolution and slice thickness, the birefringence value has been reported 2–4 × 10^−4^ in the corpus callosum of the rodent brains using either PS-OCT or polarization microscopy ^37,38^. The ability to quantify myelin content in tens to hundreds of cubic centimeters of human brain tissue promises to advance our understanding of the mechanism of various neurodegenerative diseases.

Our method was able to map fiber networks in the human brainstem, one of the most complex in the human brain, in which small fiber tracts form an intricate 3D network ^39^. It is worth emphasizing that our method can be applied to quantitatively image the 3D axis orientation and true birefringence in other fibrous tissues as well, such as cartilage ^21^, capsular ligament ^40^, enamel ^41^, uterus ^42^ and heart ^23^. Characterizing the 3D fiber organization in these tissues will play an essential role in understanding the normal functions and the pathological impact in diseases.

One limitation of the current study is that we applied our method on the *en-face* orientation data that integrated the information along the depth, assuming a constant axis of fibers bundles within a small volume intersected by the two beams. In future work, we will develop methods to extract local birefringence and orientation ^21,43–45^ to further obtain depth-resolved 3D axis orientation. We also plan to develop an automatic serial PS-OCT system with two beam illuminations for large-scale *ex vivo* human brain imaging. The combination of quantitative 3D axis orientation and serial sectioning PS-OCT opens new avenues towards imaging connectional anatomy in the healthy and diseased brain at unprecedented scales.

## Methods

### Human brain samples

We used samples excised from two neurologically normal brains from the Massachusetts General Hospital (MGH) Autopsy Suite for this study (age at death: 70 years and 46 years; 1 male and 1 female) and one neurologically normal brainstem sample from a subject enrolled in the Université de Tours body donation program (age at death: 76 years, male). Before death, the participants provided written proof of their willingness to become body donors and consented to the use of their bodies for educational or research purposes. The post-mortem intervals did not exceed 24 hours. The brains were fixed by immersion in 10% formalin and were cut into smaller blocks. We cut two blocks in the corpus callosum region from one of the MGH samples and one coronal slab from the other MGH sample. We applied refractive index matching to all the samples by incubations in 2,2-thiodiethanol solutions as described in our previous work ^27^. The *ex vivo* imaging procedures are approved by the Institutional Review Board of the MGH.

### System, data acquisition and analysis

We used a custom-built automatic serial sectioning PS-OCT (as-PSOCT) system ^15,16^ to validate our 3D orientation method (Fig. 1a). The system consists of a spectral domain PS-OCT centered at 1300 nm, motorized xyz translational stages, and a vibratome for tissue sectioning. The PS-OCT is a free-space system. A quarter wave plate is placed in the sample arm to ensure circularly polarized light on the sample. Using a Jones matrix formalism, the retardance and the optic axis orientation are obtained by the amplitude and the phase of the complex signals, respectively, on the two polarization channels. Automatic imaging and sectioning of brain blocks is controlled by custom-built software for coordinating data acquisition, xyz stage translation, and vibratome sectioning. The samples were first flat faced with the vibratome and then imaged using a scan lens (OCT-LSM3, Thorlabs, Newton, NJ). The range of imaging depth was 2.6 mm with an axial resolution of 4.2 μm in tissue.

We imaged the sample with normal illumination and illumination tilted by 15 deg about the y-axis (Fig. 1a). To implement the tilted illumination, we either mounted the sample on a rotation stage or incorporated a rotation stage into the PS-OCT scan head. The physical effect of tilting the beam or the sample is the same ^20^. For the normal illumination, one volumetric acquisition covered a field of view (FOV) of 3.5 mm × 3.5 mm. The FOV was 3.5 mm × 2 mm for the 15 deg tilted illumination. Automatic tile-scan was used to cover the whole sample, with a 20% overlap between tiles in each direction of the raster scan. For volumetric imaging of large-scale samples, a 150 μm thick slice was removed from the tissue surface by the vibratome to expose the deeper region until the whole volume was imaged.

We obtained measurements of the apparent birefringence and the optic axis orientation from the two illumination angles. The apparent birefringence was calculated by performing a linear regression of the retardance profile along 150 μm in depth and extracting the slope of the linear fit. *En-face* optic axis orientation images were determined by the peak of a histogram formed by binning the measured orientation values into 5 deg intervals along 150 μm depth. The apparent birefringence and the *en-face* orientation data were then used to compute 3D axis orientation and true birefringence.

### Image registration between the normal and titled illuminations

We applied a demon affine registration algorithm ^46,47^ to register the images between the normal and titled illumination incidences. We first registered the apparent birefringence maps from the two incidences and obtained a transformation field. Then we applied this transformation field to register the optic axis orientation maps.

### Formulation of 3D orientations in normal and tilted spherical coordinate system

To estimate the 3D optic axis via two illuminations, we define a birefringence vector $\text{Δ}n=\text{Δ}n{\left[\begin{array}{ccc} o_{x} & o_{y} & o_{z} \end{array}\right]}^{T}$, where the magnitude Δn is the true birefringence value and the unit vector ${\left[\begin{array}{ccc} o_{x} & o_{y} & o_{z} \end{array}\right]}^{T}$ represents the 3D orientation. The in-plane (Θ) and through-plane (α) orientations are obtained by vector calculus of ${\left[\begin{array}{ccc} o_{x} & o_{y} & o_{z} \end{array}\right]}^{T}$ in spherical coordinates. Let p be the unit vector parallel to the illumination beam. We use two illumination beams, one along the z-axis, $p_{1}={\left[\begin{array}{ccc} 0 & 0 & 1 \end{array}\right]}^{T}$, and one rotated about the y-axis by Ω, $p_{2}={\left[\begin{array}{ccc} sin\text{Ω} & 0 & cos\text{Ω} \end{array}\right]}^{T}$. We obtain two measurements of the apparent optic axis orientation ${\text{Θ}}_{1}$ and ${\text{Θ}}_{2}$, and two measurements of the apparent birefringence $\Delta n_{1}^{\prime }$ and $\Delta {n^{\prime }}_{2}$. This yields an apparent birefringence vector $\text{Δ}{n^{\prime }}_{1}=\text{Δ}{n^{\prime }}_{1}{\left[\begin{array}{ccc} cos{\text{Θ}}_{1} & sin{\text{Θ}}_{1} & 0 \end{array}\right]}^{T}$ under the normal illumination $p_{1}$ (Fig. 1b, left panel) and $\text{Δ}n_{2}^{\prime }=\Delta n_{2}^{\prime }{\left[cos\text{Ω}cos{\text{Θ}}_{2}\;sin{\text{Θ}}_{2}\;-sin\text{Ω}cos{\text{Θ}}_{2}\right]}^{T}$ under the tilted illumination $p_{2}$ (Fig. 1c, left panel). It is noted that the apparent birefringence values in each of the two frames are related to the true birefringence ^48^ as $\text{Δ}n_{1}^{\prime }=\text{Δ}nsin^{2}{\text{Ψ}}_{1}$ and $\text{Δ}{n^{\prime }}_{2}=\text{Δ}nsin^{2}{\text{Ψ}}_{2}$, where ${\text{Ψ}}_{1}$ and ${\text{Ψ}}_{2}$ are the polar angles (π/2−α) in the two illumination frames.

We define an estimated birefringence vector $\tilde{\text{Δ}n}$, which in turn defines its estimated in-plane orientations and polar angles, $\tilde{{\text{Θ}}_{1,2}}$ and $\tilde{{\text{Ψ}}_{1,2}}$, respectively, in the two frames. Therefore, the estimated apparent birefringence vector $\tilde{\text{Δ}n_{1}^{\prime }}$ under the normal illumination $p_{1}$ can be written as,

(2) $$\tilde{\text{Δ}n_{1}^{\prime }}=\tilde{\text{Δ}n}sin^{2}\tilde{{\text{Ψ}}_{1}}\left[\begin{array}{c} cos\tilde{{\text{Θ}}_{1}}\\ sin\tilde{{\text{Θ}}_{1}}\\ 0 \end{array}\right]=\left(\tilde{\text{Δ}n}-\frac{p_{1}^{T}\cdot \tilde{\text{Δ}n}}{{\left|p_{1}\right|}^{2}}p_{1}\right)\sqrt{1-\frac{{\left(p_{1}^{T}\cdot \tilde{\text{Δ}n}\right)}^{2}}{|\tilde{\text{Δ}n}{|}^{2}}}.$$

where the expression in the parenthesis projects the birefringence vector into the plane orthogonal to $\tilde{\text{Δ}n}$, thereby scaling it by $\sin\tilde{{\text{Ψ}}_{1}}$. The square root adds the required second $\tilde{{\text{Ψ}}_{1}}$. Similarly, the estimated apparent birefringence vector $\tilde{\text{Δ}n_{2}^{\prime }}$ under the tilted illumination $p_{2}$ can be written as,

(3) $$\tilde{\text{Δ}n_{2}^{\prime }}=\tilde{\text{Δ}n}sin^{2}\tilde{{\text{Ψ}}_{2}}\left[\begin{array}{c} cos\text{Ω}cos\tilde{{\text{Θ}}_{2}}\\ sin\tilde{{\text{Θ}}_{2}}\\ -sin\text{Ω}cos\tilde{{\text{Θ}}_{2}} \end{array}\right]=\left(\tilde{\text{Δ}n}-\frac{p_{2}^{T}\cdot \tilde{{\text{Δ}}_{n}}}{{\left|p_{2}\right|}^{2}}p_{2}\right)\sqrt{1-\frac{{\left(p_{2}^{T}\cdot \tilde{\text{Δ}n}\right)}^{2}}{|\tilde{\text{Δ}n}{|}^{2}}}.$$

### Strategy of xy-axis swapping for robust 3D axis estimation

The above computational model works well for the majority of the fiber orientations. However, a problem arises when applying Eqs. 1 – 3 to the fibers with in-plane orientations running along the y-axis (i.e., ${\text{Θ}}_{1}\approx {\text{Θ}}_{2}\approx -90/90\deg$). Since the optic axis measurement ranges between −90 deg and 90 deg, the distribution of the noise at Θ≈−90/90deg forms a U-shape (bounded by −90 deg and 90 deg, see details in Fig. S1), which deviates from the close to normal distribution of the noise at intermediate angles in Eq. 1. Consequently, the computational estimation is sub-optimal. To overcome this problem, we interchanged the x-axis and y-axis for those fibers in post-processing, to effectively convert the optic axis orientation to close to 0 deg. As a result, the noise distribution is returned back to an approximately normal one. The tilted illumination in the swapped xy-axis is equivalent to tilting about x-axis by Ω, which yields the apparent birefringence vector as $\text{Δ}n_{2}^{\prime }=\text{Δ}n_{2}^{\prime }[\text{cos}\;{\text{Θ}}_{2}\;cos\Omega \text{sin}{\text{Θ}}_{2}\;sin\Omega \text{sin}{\text{Θ}}_{2}{]}^{T}$. Correspondingly, the estimated apparent birefringence vector is written as,

(4) $$\tilde{\text{Δ}n_{2}^{\prime }}=\tilde{\text{Δ}n}sin^{2}\tilde{{\text{Ψ}}_{2}}\left[\begin{array}{c} \text{cos}\tilde{{\text{Θ}}_{2}}\\ cos\Omega \text{sin}\tilde{{\text{Θ}}_{2}}\\ sin\Omega \text{sin}\tilde{{\text{Θ}}_{2}} \end{array}\right]=\left(\tilde{\text{Δ}n}-\frac{p_{3}^{T}\cdot \tilde{\text{Δ}n}}{{\left|p_{3}\right|}^{2}}p_{3}\right)\sqrt{1-\frac{{\left(p_{3}^{T}\cdot \tilde{\text{Δ}n}\right)}^{2}}{|\tilde{\text{Δ}n}{|}^{2}}},$$

where $p_{3}={\left[\begin{array}{ccc} 0 & -sin\Omega & cos\Omega \end{array}\right]}^{T}$ is the new direction vector of the tilted illumination beam about the x-axis. Therefore, we combine the two sets of equations and adapt a criterion that Eqs. 1, 2, and 3 are used to retrieve fibers with optic axis $\left|{\text{Θ}}_{1}\right|$ < 45 *deg* and Eqs. 1, 2, and 4 are used to estimate fibers with optic axis $\left|{\text{Θ}}_{1}\right|$ ≥ 45 *deg*.

### Optimization of 3D orientation

The optimization algorithm takes the registered *en-face* orientation (${\text{Θ}}_{1}$ and ${\text{Θ}}_{2}$) and apparent birefringence ($\Delta n_{1}^{\prime }$ and $\Delta {n^{\prime }}_{2}$) from normal and tilted incidences as inputs, converts them into the birefringence vector $\text{Δ}n_{1}^{\prime }$ and $\text{Δ}{n^{\prime }}_{2}$, and outputs the estimated Δn, which yields the true birefringence value Δn and the 3D orientation vector ${\left[\begin{array}{ccc} o_{x} & o_{y} & o_{z} \end{array}\right]}^{T}$ (see details in previous section). The 3D orientation vector is further converted into in-plane and through-plane orientations for better visualization. We used the function *fminsearch* in Matlab (Mathworks, Natick, MA) to minimize the difference between the estimated and the measured birefringence vectors. The initialization values were set to be $[\begin{array}{ccc} 0, & 0 & ,0 \end{array}]$ and the optimization stopped based on an error tolerance of 1e-20 in the *fminsearch* function.

### Quantifying fiber orientation distribution

To quantitatively analyze the orientations obtained from the uniform corpus callosum sample, we constructed fiber orientation distributions (FODs) by generating histograms of the orientation map in every 0.5 mm × 0.5 mm region using a bin width of 5 deg. We found the primary peak and full width at half maximum (FWHM) of each FOD, then calculated the averaged peak direction and FWHM over the FODs in a 2.5 mm × 2.5 mm or 3 mm × 3 mm region of interest (ROI). Pixels with low birefringence values were excluded from the analysis.

### Microscopic tractography

Tractography was generated for the volumetric brainstem data, using a DTI model in Diffusion Toolkit ^49^. The estimated 3D axis orientation was used to track the fiber using the streamline algorithm with an angular threshold of 60 degree and no threshold on the intensity. Due to the high resolution of serial sectioning PS-OCT, fiber tracking on the full 3D data would generate an extremely large data size beyond the ability of any visualization tools. As a result, we manually segmented four white matter structures in both brainstem hemispheres and performed tractography on each segmented structure of interest. The tracts were visualized in TrackVis (Version 0.6.1; trackvis.org).

### Diffusion weighted MRI and tractography

The coronal slab sample was packed in fomblin and scanned in a small-bore 4.7 T Bruker BioSpin MRI scanner (maximum gradient strength 660 mT/m) using a diffusion-weighted 3D echo-planar imaging sequence with the following parameters: 0.25 mm isotropic resolution, TR = 500 ms, TE = 47.8 ms, 20 segments, 5 averages, δ=15ms, Δ=19ms, b-value = 4,000 s/mm^2^, one b=0 volume and 12 volumes with non-colinear gradient directions. After denoising ^50^ and correction for eddy-current distortions ^51^, deterministic tensor tractography ^29^ was performed, seeding in every white-matter voxel.

The brainstem sample was packed in fombin and scanned in a 3T Siemens Trio MRI scanner using a diffusion-weighted SSFP sequence with the following parameters: 0.3 mm isotropic resolution, TR = 31.19 ms, TE = 25.6 ms, flip angle = 35°, bandwidth = 149 Hz/Px, b-value = 20,000 s/mm^2^, one b=0 volume and 30 gradient directions. Diffusion tensor imaging (DTI) model was used to extract the fiber orientation.

## Data Availability

All data included in this study are presented in the figures. Raw image data of the human brain may be obtained from the corresponding author following MGH guideline.
